# Validation of the Equine Behaviour Assessment and Research Questionnaire (E-BARQ): A New Survey Instrument for Exploring and Monitoring the Domestic Equine Triad

**DOI:** 10.3390/ani10111982

**Published:** 2020-10-28

**Authors:** Kate Fenner, Sarah Matlock, Jane Williams, Bethany Wilson, Andrew McLean, James Serpell, Paul McGreevy

**Affiliations:** 1 Sydney School of Veterinary Science, University of Sydney, Camperdown, NSW 2006, Australia; bethany.wilson@sydney.edu.au (B.W.); paul.mcgreevy@sydney.edu.au (P.M.); 2 Equine Sciences Program, Colorado State University, Fort Collins, CO 80523, USA; sarah.matlock@colostate.edu; 3 Equine Department, Hartpury University, Gloucester GL19 3BE, UK; jane.williams@hartpury.ac.uk; 4 Equitation Science International, 3 Wonderland Ave, Tuerong, VIC 3915, Australia; andrewmclean@esi-education.com; 5 School of Veterinary Medicine, University of Pennsylvania, Philadelphia, PA 19104, USA; serpell@vet.upenn.edu

**Keywords:** behaviour assessment, horse behaviour, horse welfare, rider safety, domestic equine triad, ethical equitation

## Abstract

**Simple Summary:**

The Equine Behaviour Assessment and Research Questionnaire (E-BARQ) is an online questionnaire collecting information from horse-owners and riders on their horses’ training, management and behaviour. In order to compare different populations of domestic equids—for example, according to their breed, discipline, training or management type—it is important that the questionnaire administered to participants has been validated and found to be reliable. The questionnaire must reflect a true assessment of the horse’s behaviour, and, when taken by different users or the same user over time, the results should remain consistent. This article describes the process of validating the E-BARQ questionnaire, using three separate procedures. The results confirmed that the E-BARQ represents a standardised and validated behavioural assessment tool for horses.

**Abstract:**

The Equine Behaviour Assessment and Research Questionnaire (E-BARQ) was developed to obtain quantitative data on the domestic equine triad: training, management and behaviour. It can be taken repeatedly, thus collecting longitudinal data to enable evaluation of how changes in a horse’s training and management are reflected in that horse’s behaviour over time and how these changes can impact horse welfare in the longer term. Questionnaire validation and reliability were tested by determining (a) whether an owner’s subjective ratings of their horse’s problematic behaviours or undesirable temperament traits were reflected in the questionnaire scores obtained for that horse (construct validity), (b) whether two respondents, equally familiar with a particular horse, reported comparable scores for that horse through the questionnaire (inter-rater reliability), and (c) whether the same respondent, scoring the same horse after a known interval of time, recorded similar responses (intra-rater reliability). Construct validity testing of 1923 responses showed significant alignment between owners’ reported experience of focal horses’ behaviour and those horses’ E-BARQ scores, with scores varying from 1.13 to 1.34 for ridden horse behaviour (all *p* < 0.001) and from 1.06 to 1.43 for non-ridden horse behaviour (all *p* < 0.001). Inter-rater reliability testing of ten horse–rider pairs revealed that 203 of the 215 question items were significantly aligned (*p* < 0.001) when tested by two independent raters. Of the remaining 19 items, four had fair alignment (ĸ = 0.174–0.316; *p* = 0.281) and ten items, largely related to whether the horse shows behavioural signs related to anxiety when taken away from home, did not align (ĸ = 0; *p* = 1). Intra-rater reliability tests showed that the responses significantly aligned on all 215 question items tested (*p* < 0.001). The results of these tests confirmed the construct validity and reliability of E-BARQ as a standardised behavioural assessment tool for horses.

## 1. Introduction

Behavioural problems (here encompassing behaviours that horse riders, owners and caregivers consider undesirable or dangerous) compromise rider and handler safety and can jeopardise horse welfare. Stereotypic and redirected behaviours have long been associated with suboptimal management practices [1]. However, non-stereotypic behavioural problems, including unwelcome responses in-hand and under-saddle, are often encountered by horse riders and handlers, with a reported 28% of the 200,000 horses in the United States being relinquished to rescue organisations for this reason [2]. Normando et al. [3] found links between riding style and reported behavioural problems, including stereotypies. Specifically, they found associations between English-style riding and restrictive stabling practices, also associated with more frequently reported locomotion stereotypies. Hockenhull and Creighton [4] found that 91% of UK horse owners reported ridden behavioural problems with their horses, among which shying was the most frequently cited behaviour [4]. Hockenhull and Creighton [5] also identified risk factors associated with stable-related and handling behavioural problems when investigating horse management practices. Risk factors included confinement in stables, turn-out schedules, social isolation and the amount of time that owners spent with their horse each day. These researchers also revealed that training styles and equipment were risk factors for ridden behavioural problems, including failing to slow or jump and extreme conflict such as bucking, bolting and rearing [6].

Understanding how and why problem behaviours develop will advance safety and welfare. Horse owners and riders have an ethical obligation to be aware of how their training affects their horse because equitation largely relies on the appropriate use of pressure during negative reinforcement [7], the application of aversive stimuli (usually pressure applied via the rider’s hands and legs) until the horse offers the desired response, at which point the pressure must be removed to reinforce conditioning of the correct response [8]. The use of prolonged or excessive pressure is contraindicated because it leads to habituation (an outcome which horses are especially prone to) and the consequent need for more pressure in future [9].

When problems manifest, handlers and riders often find themselves in a downward spiral of punishment and increasingly dangerous behaviour [10,11]. While ethical equitation employs combined reinforcement, riding the horse obliges the use of negative reinforcement with seat, leg and rein signals. Horses, as prey animals vulnerable to attack when injured or distressed, are easily habituated to aversive stimuli, becoming non-responsive [12]. Handlers and riders can then resort to punishment when the horse fails to respond to applied stimuli.

In domestic animals, there is a triadic relationship between training, management and behaviour. For equids, when training and management flaws are left unresolved, the horse’s deteriorating behavioural responses can result in suboptimal care and welfare. For example, the horse that has been confined to a stable for an extended period is likely to express post-inhibitory rebound locomotory behaviour [13,14] in the form of bolting and bucking. This may unseat the rider and, as a result, prompt the use of inappropriate punishment [14]. While considerable research has focused on the training of horses [15,16,17,18,19,20,21,22,23,24,25,26] and management [3,4,27,28,29,30,31,32,33,34,35,36], a deeper understanding of how the triadic elements interact is now required to improve horse welfare and advance ethical equitation. By gathering baseline data on the domestic equine triad, researchers will be able to define what constitutes normal behaviour for horses and identify best-practice interventions according to an evidence base.

Horses vary widely in their responses to various types of standardised behavioural testing based on equine and equitation science [37]. Furthermore, researchers using behavioural tests to assess temperament or reactivity often study cohorts of horses that have been previously exposed to diverse management and training regimes. These diverse regimes could contribute variably to the ontogeny of the behaviours observed [9]. Small nuanced changes in behaviours can be reliably observed and recorded by animal-owners and caregivers [38], and the use of online surveys to gather such data has transformed how research is conducted across many industries, from science to marketing [39]. To define what constitutes normal equine behaviour, there is a clear need for an accessible, objective, standardised and validated data-collection tool, especially one that facilitates comparison between horse populations [40].

There is a paucity of research on the ontogeny and epidemiology of behavioural problems in horses. Published studies in this domain have typically been geographically specific and have investigated particular behavioural attributes without the benefit of baseline behavioural data [41], such as reports on how independent the horse appears, how it interacts with other horses and humans and how responsive it is to signals and cues. Many studies also suffer from small sample numbers, making generalisation across populations difficult. There are several reasons for this, the main one being that horses are large and expensive, thus costly to incorporate into experimental designs that impose uniform management on experimental subjects. For this reason, as has been done with other species [42,43], low-cost equine studies often gather data from horses in their home environment and harvest behavioural observations reported by their owners and caregivers.

To understand how and why behaviours, particularly those that can jeopardise rider or handler safety and horse welfare, develop and persist over time, large-scale data collection and analyses are required. To date, data collection in the area of horse behaviour has rarely taken a holistic approach, investigating training, management and behaviour at the same time, and nor has longitudinal data collection been a priority.

To optimise questionnaire reliability, owners’ reports must focus on the frequency of observations, negating the need for participants to explain the behaviour or comment on the horse’s motivations for performing behaviours [41]. Historically, owner-sourced data on horse temperament often required respondents to interpret behaviour. For example, questions that derive such data related to how “anxious” a horse is in a given environment require owners to observe behaviours, decipher which are relevant and, finally, score the behaviour without a reference point to benchmark its current frequency against [44]. Individuals likely differ in how they interpret behaviour and the assumptions they make about what motivates the horse to behave in a particular way [45,46], thus compromising survey reliability.

Such limitations on data collection led to the development of the Equine Behavior Assessment and Research Questionnaire (E-BARQ), a sister project to both the Canine Behavioral Assessment and Research Questionnaire (C-BARQ), [43] for dogs and the Feline Behavioral Assessment and Research Questionnaire (Fe-BARQ) [42] for cats. The C-BARQ, launched in 2005, has collected data on over 85,000 dogs and been used in more than 100 published studies. The Fe-BARQ has been collecting data on the behaviour of domestic cats since 2016 and currently has over 7000 cats reported through the database. E-BARQ provides participants with the opportunity to return to the questionnaire and update their responses, a feature that offers researchers longitudinal data. This is arguably a critical feature when investigating horse behaviour as horses change owners and disciplines more regularly than their canine or feline counterparts.

The interaction between training, management and behaviour has been labelled the domestic equine triad [41]. Providing researchers and the equine industry with a reliable, standardised and validated behavioural assessment tool, that collates and processes their own data across the domestic equine triad, will improve horse rider and handler safety, and horse welfare. However, the importance of demonstrating reliability cannot be overlooked. The tool must be shown to describe what it purports to measure, it must show consistency when repeated, and finally, it must allow different participants to score focal horses in a similar way. For the survey instrument to be widely accessible, relevant and reliable, the tests described in this article are required.

The aim of the current study was to validate the E-BARQ questionnaire as a reliable, standardised behavioural assessment tool for horses. To demonstrate the validity of the E-BARQ as a behavioural assessment instrument, we undertook three testing protocols [47]. Construct validity, demonstrating that the E-BARQ was measuring what it set out to measure, was evaluated by comparing owners’ subjective assessment of their horses’ behaviour with the detailed scores obtained from the questionnaire. Inter-rater reliability was evaluated by comparing rider pairs’ scores for the same horse. Finally, intra-rater reliability was assessed by comparing the scores given by the same assessor to the same horse over time.

## 2. Materials and Methods

### 2.1. Participant Recruitment

Survey responses from 1923 E-BARQ questionnaires were used to test construct validity. The survey had been widely distributed with respondents from 33 countries, with 60% pure bred horses comprising more than 78 different breeds, 58% geldings and 38% mares (with the remainder stallions and colts both <1% and fillies <2%). More than 80% of owners reported working with or keeping horses before the age of 16 and 83% reported having more than 8 years of riding or horse handling experience. Most participants (90%) were in the age range of 18 to 64 years.

Participants for both the inter- and intra-rater reliability tests were recruited via two university equine departments: Hartpury University, United Kingdom and Colorado State University, USA. To be eligible for inclusion within inter-rater reliability testing, participants were required to be over 18 years of age and capable of the equivalent of British Horse Society Stage 3 riding ability, be equally familiar with the horse under saddle and on the ground and should have completed the questionnaire independently but within one week of each other. To be eligible for inclusion within intra-rater reliability testing, participants were required to own or have access to a horse with which they had prior knowledge and experience, been currently working with the horse under saddle and/or on the ground and should have completed the questionnaire twice, approximately 30 days apart. This study was conducted under the approval of the University of Sydney Human Research Ethics Committee (approval number: 2012/656).

### 2.2. Questionnaire Validation

#### 2.2.1. Construct Validity

A previous rotated principal component analysis revealed 55 rotated components in the E-BARQ questionnaire [48]. These components were used to assess owner-reported behaviours (see Supplementary File S1 for a full list of items).

Respondents were asked, “Have you been experiencing problems with your horse’s behaviour?” in the past six months. Answer options were “no problems”, “only minor problems”, “moderate problems” or “serious problems” (see Supplementary File S1 for the full E-BARQ questionnaire).

To test the validity of the questionnaire, respondents’ answers to “Have you been experiencing problems with your horse’s behaviour?” were compared to their horses’ scores on the E-BARQ items [42,43]. We expected to see horses belonging to respondents who reported not having problems with behaviour to score lower (fewer problems) than those who did report having problems with their horses’ behaviour.

#### 2.2.2. Inter-Rater Reliability

We surveyed 10 pairs of riders, each equally familiar with a particular horse, to determine whether they reported the same behaviours in that horse. The 20 respondents, assigned to pairs, reported on 10 individual horses. To test the inter-rater reliability of the questionnaire, we compared the scores provided by the rider pairs by calculating the Cohen’s Kappa for scores awarded by the two raters.

#### 2.2.3. Intra-Rater Reliability

We surveyed 52 riders, each on one focal horse, and re-surveyed them 30 days later. We calculated Cohen’s Kappa for the two scores awarded by the same rider, expecting to see significant similarities.

### 2.3. Statistical Analysis

#### 2.3.1. Construct Validity

Construct validity was assessed using the following:

- The E-BARQ contains items related to both horse and owner demographics, management and behaviour. One hundred and thirty-four E-BARQ items that related to behaviour and 50 items that related to maintenance and gear were selected for construct validity testing (see Supplementary File S1 for the full question item text).
- A tabulated matrix was used, with six response options from “never” to “always” and “not applicable/observed”, to ask respondents how often their horses performed each behaviour. Items were either positively or negatively framed. For example, “how often does your horse buck” (negatively framed) and “does your horse load on to the trailer first time” (positively framed). These negatively framed question items were reversed for scoring consistency (a high score equated to more undesirable behaviours).
- As all of the selected E-BARQ items were ordinal, they were treated as continuous (because not all items were measured on the same ordinal scale).
- A multinominal log-linear regression was performed on the respondents’ scores on Question 51: “Have you been experiencing problems with your horse’s behaviour in the last six months?”. An ordinal logistic regression, using the ridden horse questionnaire items, was attempted using the ordinal package. However, diagnostic testing found that the proportional log odds assumption did not hold. Thus, a multinominal log-linear model was applied using the nnet package [49]. For comparison with the ridden horse questionnaire analysis, a multinominal log-linear model was also applied to the non-ridden questionnaire respondents’ score on Question 51, “Have you been experiencing problems with your horse’s behaviour in the last six months?”, using the nnet package [49].

#### 2.3.2. Inter- and Intra-Rater Reliability

Inter-rater reliability was calculated using Cohen’s Kappa for the scores awarded by the two raters. Where respondents had actively entered “not applicable” or “not observed” as responses, and not skipped the question, their responses were retained and, when analysed as an ordinal response, were assigned an order below “never”. Responses were treated as missing where no answer was given because respondents had not indicated that the behaviour was never observed.

Intra-rater reliability was calculated using the Cohen’s Kappa for the scores awarded by the same respondent, with 30 days between assessments. As above, “not applicable” and “not observed” responses were retained and assigned an order below “never”, when analysed as an ordinal response. Responses were treated as missing where no answer was given because respondents had not indicated that the behaviour was never observed.

Cohen’s Kappa statistics were interpreted in accordance with McHugh [50] and Hohlbaum et al. [51] as follows: values ≤0 as indicating no agreement and 0.01–0.20 as none to slight, 0.21–0.40 as fair, 0.41–0.60 as moderate, 0.61–0.80 as substantial and 0.81–1.00 as almost perfect agreement.

## 3. Results

### 3.1. Construct Validity

Construct validity was assessed using respondents’ self-assessed rating of problem behaviour (see Table 1).

#### 3.1.1. Ridden Horse Questionnaire Items

**Figure 1:**
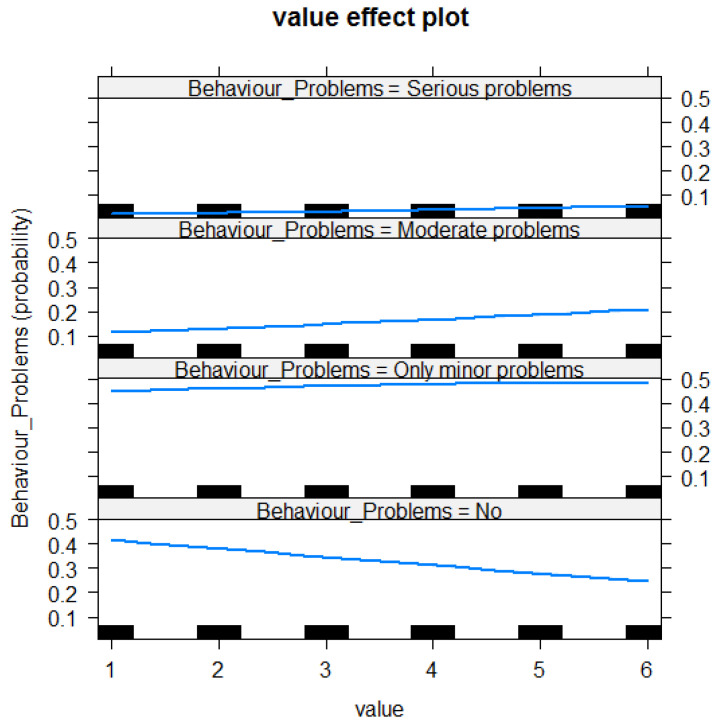
Value effect plot for the ridden horse questionnaire items showing the probability of behaviours reported by the four self-reported categories (no problems, only minor problems, moderate problems and serious problems). The more undesirable or dangerous the behaviours reported were, the higher the value scored for the horse. Value 1 represents not applicable/not observed, value 2 represents never and value 6 represents always.

For the ridden horse questionnaire items, respondents categorised their horses as having “no problems”, “only minor problems”, “moderate problems” or “serious problems”. The probability of a respondent reporting a behaviour to be frequently observed (1 = not observed/applicable, 2 = never, 6 = always) is shown in Figure 1. Respondents were more likely to score horses with minor, moderate and serious behaviour problems higher than horses with no behavioural problems across questions related to ridden behaviour (Chisq = 2587.4, 3df, <2.2e−16***, *p* < 0.001; see Table 2 and Figure 1).

#### 3.1.2. Unridden Horse Questionnaire Items

**Figure 2:**
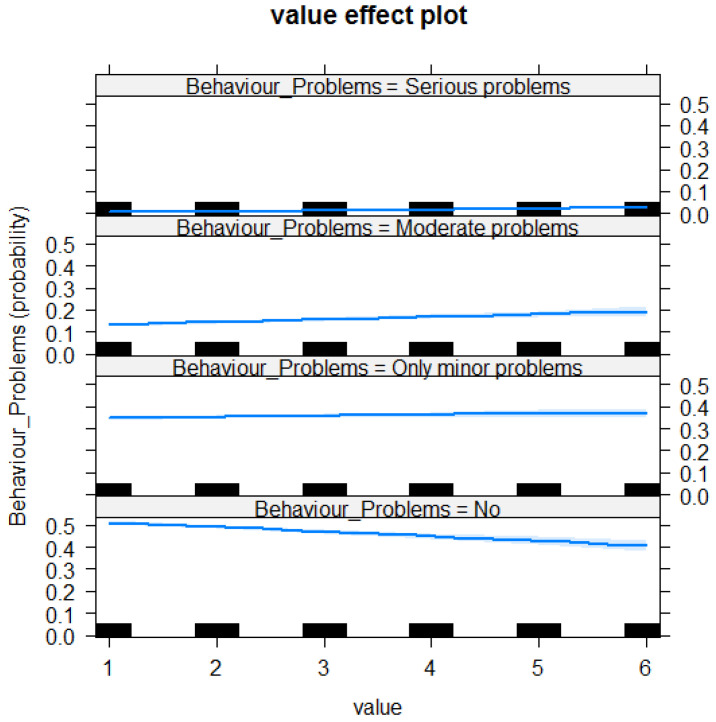
Value effect plot for the unridden horse questionnaire items showing the probability of behaviours reported by the four self-reported categories (no problems, only minor problems, moderate problems and serious problems). The more undesirable or dangerous the behaviours reported were, the higher the value scored for the horse. Value 1 represents not applicable/not observed, value 2 represents never and value 6 represents always.

For the unridden horse questionnaire items, respondents categorised their horses as having “no problems”, “only minor problems”, “moderate problems” or “serious problems”. The probability of a respondent reporting a behaviour to be frequently observed (1 = not observed/applicable, 2 = never, 6 = always) is shown in Figure 2. Respondents were more likely to score horses with minor, moderate and serious behaviour problems higher than horses with no behavioural problems across questions related to non-ridden behaviour (Chisq = 87.466, 3df, <2.2e−16***, *p* < 0.001; see Table 3 and Figure 2).

### 3.2. Inter-Rater Reliability Testing

#### Omnibus tests

An overall inter-rater reliability test was performed using all non-demographic E-BARQ items. The 215 items were obtained from the question matrices 52, 55, 57, 60, 61, 62, 63, 64, 65, 66, 68, 69, 71 and 74 of the ridden survey (see Supplementary File S2 for full item and question number list). The omnibus test results and the linear-weighted Kappa scores for individual questions are shown in Table 4 and Table 5, respectively.

### 3.3. Intra-Rater Reliability Testing Using Cohen’s Kappa Scores

#### Omnibus Test

Overall intra-rater reliability was tested using all non-demographic E-BARQ items. The 215 items were obtained from the question matrices 52, 55, 57, 60, 61, 62, 63, 64, 65, 66, 68, 69, 71 and 74 of the ridden survey (see Supplementary File S3 for full item and question number list). The omnibus test results and the linear weighted Kappa scores for individual questions are shown in Table 6 and Table 7, respectively.

## 4. Discussion

The E-BARQ performed well in the construct validity testing. Owners reporting moderate or serious problems with their horses during the six months before taking the E-BARQ scored significantly worse than those reporting no problems or minor problems. The results in Figure 1 and Table 2, the ridden horse questionnaire, demonstrate that respondents reporting “no behavioural problems” are obtaining fewer high-level behavioural problems (where 1 = N/A, 2 = never and 6 = always). In contrast, with each of the three reported problem levels (mild, moderate and serious problems), the frequency of reported problems significantly increases (see Table 2). Conversely, those reporting no problems or mild behavioural problems scored better on items relating to problem behaviours, providing reliable construct validity for the E-BARQ questionnaire. Similar results in Figure 2 and Table 3, the non-ridden horse questionnaire, demonstrate that respondents reporting “no behavioural problems” are obtaining fewer high-level behavioural problems (where 1 = N/A, 2 = never and 6 = always). In contrast, with each of the three reported problem levels (mild, moderate and serious problems), the frequency of reported problems significantly increases (see Table 3).

Horses reported to have only minor problems were by far the most common and it is unlikely that even those horses with reportedly severe problems would have poor scores in all areas of behaviour (see Supplementary File S1 for a full list of items). It is noted that this type of validation is not truly independent, because it relies on the behavioural information of the owner and not all owners are likely to perceive “problem behaviours” in the same way. Nevertheless, it is a highly relevant attribute to test for validity because behavioural problems are known to be the biggest risk to the welfare of the pleasure riding horse [4,52].

When interacting with almost all items within the E-BARQ, respondents report on the frequency of behavioural observations and no interpretation of behaviour is required. The current inter-rater reliability testing demonstrates that the E-BARQ encourages this style of reporting consistently, suggesting that E-BARQ results are generalisable to disparate populations of horses. Among the Cohen’s Kappa scores for the 215 items, 33 scored more than 0.860 (almost perfect agreement), 40 scored <0.646 (substantial agreement), 123 scored <0.416 (moderate agreement), five scored <0.4 (fair agreement), four scored 0.361 (slight agreement), and ten, relating to the horse showing anxiety when away from home, showed no agreement with a score of zero (see Supplementary File S2 for a full list of question items and Kappa alignment scores).

No agreement was found between the ten items relating to the horse showing signs of anxiety when away from home (ĸ = 0) (see Supplementary File S2). The behaviours that could be reported in this part of the E-BARQ included restlessness, pacing, vocalising, bucking, rearing and moving about or pulling back when tied. When respondents reported on similar behaviours observed in the same horse when separated from other horses at home, their scores showed moderate alignment (ĸ = 0.458). This may reflect an operator effect on behaviour, in that it supports the findings of other researchers [53] that horses react differently when handlers and riders are anxious. Horses can be taken away from home for a variety of reasons, from high-level competition to pleasure riding outings, and these differences are likely reflected in the handlers’ interaction with the horse at the time. For example, a rider at a high-level competition may feel anxious themselves, changing the way they handle the horse. It may also reveal the limited experience that some participants could have had in taking the horse away from home and the different types of events attended, if they did not typically take the horse away from home. This effect, originating from the arousal level of the handler, may also apply when respondents were asked whether the horse will stand for veterinary and farrier procedures, in that only slight alignment was found. Veterinary visits can be stressful events for horse owners, particularly when the horse is unwell or requires invasive procedures, possibly making horses difficult to manage [54]. Owners of horses that have not been appropriately trained to stand for the veterinarian or farrier may also alter their handling behaviour during farrier visits as a result of their expectations [55]. Further investigations into equine manifestations of anxiety displayed at different events and venues, especially with different handlers, would be of interest.

The other notable finding here was the score of fair alignment when respondents were asked how quickly their horse learns. There were four items, including how quickly the horse learned with food rewards, positive reinforcement (other than food), pressure release and punishment/correction. With the exception of this question set, E-BARQ simply asks respondents to report on the frequency of behavioural observations. This more subjective question set was added because the authors recognised the influence that riders’ and handlers’ preconceived ideas can have on the horse–human relationship [45,46]. For example, if a rider believes the horse is being stubborn, rather than confused, they may become more reactive and thus more likely to punish or correct the horse. Further studies should explore how the interpretations of videos of a focal horse undertaking a task differ when viewed by riders and handlers with different beliefs about how well the horse learns.

Intra-rater reliability testing revealed very good agreement between ratings. Interestingly, none of the intra-rater associations were within the perfect or near perfect range, presumably because horse behaviour changes over time. However, of the 215 items assessed, only four scored a Cohen’s Kappa lower than 0.485 (moderate agreement). These four items, scoring only 0.262 (fair agreement [50]), related to horses pulling or lagging behind when being led. It could be argued that this question set is slightly more subjective than some others in the E-BARQ questionnaire and, by asking the respondent to interpret “pulling” or “lagging”, may introduce inconsistencies within respondents. Furthermore, as leading is a frequent activity when handling horses, these particular items may be more subject to recall bias than others. For example, if a horse that does not normally pull on the lead suddenly does so one day, close to the respondent taking an E-BARQ, the respondent is likely to remember the recent unwelcome behaviour. Other behaviours, such as bucking or problematic loading onto a trailer, are less frequently encountered and, presumably, are rarely forgotten. Such behaviours are also more objectively assessed, not requiring the handler to interpret the horse’s behaviour. Further research, comparing larger numbers of E-BARQ results over longer periods, would be of interest in this area.

Again, interestingly, E-BARQ has, arguably, only four items in its subjective question set that explore how quickly the horse learns from combined reinforcement. This set scored 0.555 (moderate agreement) when assessed with the intra-rater reliability tests. The same items scored 0.174 (slight agreement) when different riders assessed a focal horse. This finding underlines the need for questions to be objective and to avoid respondents having to interpret behaviour, as suggested by Fenner et al. [41].

The current study did have some limitations. Participation was voluntary and, as a result, the results may be exposed to inherent subject and response bias and could over-represent views of horse owners who engage with online platforms. Some measures of performance are open to individual interpretation and, as discussed, can introduce subjectivity into survey responses—for example, personal assessment of a horse’s response to the rider’s cues. Our results suggest that intra- and inter-reliability is good for almost all questions within the E-BARQ survey across two sampling periods and between respondents. However, inter- and intra-rater reliability testing with larger sample sizes and across different geo-cultural areas would be advantageous.

## 5. Conclusions

These validation tests show the E-BARQ to be a reliable, consistent and valid questionnaire for assessing behaviour in horses. This instrument will reveal what constitutes normal behaviour in horses, help to identify those training and management practices that lead to a deterioration in behaviour and, in the future, become a valuable predictive tool for the development of problematic behaviour. Thus E-BARQ has the potential to collate evidence that could greatly improve rider safety and horse welfare, leading to evidence-based practice and ethical equitation.

## Figures and Tables

**Table 1:** The number of respondents reporting behavioural problems in each category. A total of 1923 respondents were asked whether “you have been experiencing problems with your horse’s behaviour in the last six months?”.

Questionnaire	No Problems	Only Minor Problems	Moderate Problems	Serious Problems
Ridden horse	578	745	228	51
Unridden horse	158	111	48	4
Total	736	856	276	55

**Table 2:** The probability of three levels of undesirable behaviours being reported, when compared to no behavioural problems, in the self-reported categories on the ridden horse questionnaire. The more undesirable or dangerous the behaviours reported were, the higher the value scored for the horse.

Reported Problem Level	Estimate	Standard Error	Statistic	*p*-Value
No behavioural problems	Reference			
Only minor problems	1.13	0.00386	30.8	<0.001
Moderate problems	1.25	0.00508	43.8	<0.001
Serious problems	1.34	0.00879	33.2	<0.001

**Table 3:** The probability of undesirable behaviours being reported, when compared to no behavioural problems, in the self-reported categories of the unridden horse questionnaire. The more undesirable or dangerous the behaviours reported were, the higher the value scored for the horse.

Reported Problem Level	Estimate	Standard Error	Statistic	*p*-Value
No behavioural problems	Reference			
Only minor problems	1.06	0.0139	4.22	<0.001
Moderate problems	1.13	0.0178	6.86	<0.001
Serious problems	1.43	0.0484	7.36	<0.001

**Table 4:** Inter-rater reliability omnibus tests. Cohen’s Kappa scores matrix containing multiple individual items. The table shows Kappa scores using linear weighted Kappa only. Paired responses represent the number of paired responses from which the Kappa was calculated. They include questions × the number of horses (possible question pairs for comparison) minus “true” missing values. Supplementary File S2 contains the 215 question items in each matrix.

Weights	Paired Responses	Raters	Kappa	Z Statistic	*p*-Value
Unweighted	2013	2	0.559	50.1	<0.001
Equal	2013	2	0.667	45.6	<0.001
Squared	2013	2	0.727	32.9	<0.001

**Table 5:** Inter-rater reliability results. Each Equine Behavior Assessment and Research Questionnaire (E-BARQ) question listed is a matrix containing multiple individual items. Supplementary File S2 shows question numbers and question text for each matrix. The table shows Kappa scores using linear weighted Kappa only.

Question	Kappa	Z Statistic	*p*-Value
52: Frequency of training type and rider gender	0.713	12.8	<0.001
55: Stand for maintenance procedures	0.361	4.25	<0.001
57: Acceleration, deceleration, canter leads, buck, rear, bolt, toss head	0.591	15.4	<0.001
60: Responsiveness to cues	0.416	8.03	<0.001
61: Behaviour when leading	0.665	6.07	<0.001
62: Speed of learning	0.174	1.27	0.281
63: Tack worn	0.918	15.7	<0.001
64: Shy away from novel objects	0.516	8.8	<0.001
65: Defensive or aggressive behaviour	0.458	11.1	<0.001
66: Behaviour when separated from others	0.595	7.74	<0.001
68: Behaviour when taken away from home	0	0	1
69: Stereotypies and repetitive behaviours	0.597	12.7	<0.001
71: Maintenance procedures	0.860	6.58	<0.001
74: Catching, bridling, loading, travelling	0.646	13.2	<0.001

**Table 6:** Intra-rater reliability omnibus tests. Kappa scores representing the reliability of rater scores across a period of 30 days. Paired responses represent the number of paired responses from which the Kappa was calculated. They include questions × the number of horses (possible question pairs for comparison) minus “true” missing values.

Weights	Paired Responses	Raters	Kappa	Z Statistic	*p*-Value
Unweighted	8861	2	0.545	100	<0.001
Equal	8861	2	0.715	98.8	<0.001
Squared	8861	2	0.82	77.2	<0.001

**Table 7:** Intra-rater reliability results. Each E-BARQ question listed is a matrix containing multiple individual items. Supplementary File S3 shows question numbers and question text for each matrix. The table shows Kappa scores using linear weighted Kappa only.

Question	Kappa	Z Statistic	*p*-Value
52: Frequency of training type and rider gender	0.710	26.5	<0.001
55: Stand for maintenance procedures	0.626	13	<0.001
57: Acceleration, deceleration, canter leads, buck, rear, bolt, toss head	0.673	34.5	<0.001
60: Responsiveness to cues	0.485	18.5	<0.001
61: Behaviour when leading	0.262	4.89	<0.001
62: Speed of learning	0.555	11.2	<0.001
63: Tack worn	0.723	30.8	<0.001
64: Shy away from novel objects	0.513	20.5	<0.001
65: Defensive or aggressive behaviour	0.501	26.2	<0.001
66: Behaviour when separated from others	0.611	15.1	<0.001
68: Behaviour when taken away from home	0.582	9.99	<0.001
69: Stereotypies and repetitive behaviours	0.546	24.9	<0.001
71: Maintenance procedures	0.718	17.3	<0.001
74: Catching, bridling, loading, travelling	0.694	29.7	<0.001

## Supplementary Materials

The following are available online at https://www.mdpi.com/2076-2615/10/11/1982/s1, Supplementary File S1: Full list of items; Supplementary File S2: Inter-rater reliability Kappa scores for 215 question items; Supplementary File S3: Intra-rater reliability Kappa scores for 215 question items.
